# Human-Forest interfaces in Hugumburda-Gratkhassu National Forest Priority Area, North-eastern Ethiopia

**DOI:** 10.1186/s13002-018-0218-7

**Published:** 2018-02-23

**Authors:** Leul Kidane, Sileshi Nemomissa, Tamrat Bekele

**Affiliations:** 1 Department of Biology, College of Natural and Computational Sciences, Mekelle University, P.O. Box 231, Mekelle, Ethiopia; 2 Department of Plant Biology and Biodiversity Management, Addis Ababa University, P.O.Box 3434, Addis Ababa, Ethiopia

**Keywords:** Anthropogenic factors, Direct matrix ranking, Ethnobotany, Indigenous knowledge, Key informants, Ethiopia

## Abstract

**Background:**

Traditional management regimes and knowledge systems of forest resources have shaped forests throughout the world where materials from individual species are harvested in a sustainable manner. To comprehend this, the vegetation of Hugumburda-Gratkhassu Forest was described and related to anthropogenic factors.

**Methods:**

Three ethnobotanical research methods were used to collect indigenous knowledge of the local inhabitants related to conservation and utilization of forest resources. Direct matrix ranking was conducted to discover local attitudes on species preference for multiple use. During this work, the 46 most important tree and shrub species were selected based on recommendations of local guides and key informants to determine the range of uses obtained from each species. Through paired comparison, activities supposed to be the major cause of degradation of the forest were adopted. Pairs of activities were then established from the relation n (n-1)/2. Each respondent was then asked to select an activity that he considered being a major problem to management of the forest. Semi-structured interviews were used to obtain information from sixty local informants to address community attitudes towards forest management and utilization.

**Results:**

The result obtained from direct matrix ranking showed; that 20 out of 46 plant species compared had the highest scores and rank, indicating that these species are the most important and are exploited by the local communities for multiple purposes. The paired comparison exercise revealed logging for construction materials to be the major threat to the forest due to cutting of large volume of wood for construction of churches, health centers, schools and new houses. *Juniperus procera, Olea europaea ssp. cuspidata, Rhus glutinosa, Ficus sur, Hagenia abyssinica, Cassipourea malosana* and *Acacia etbaica* were the most selected and exploited plant species for these purposes.

**Conclusions:**

Survival of protected areas depends on the support of local communities, rather than on fences, fines, or even force. The local communities in the study area have a rich indigenous ecological knowledge to suggest appropriate solutions for improvement of the forest resources. Thus the old tradition of isolating forests from the community has to be avoided and the basic needs and traditional rights of the communities over the uses of forest resources should be recognized.

**Electronic supplementary material:**

The online version of this article (10.1186/s13002-018-0218-7) contains supplementary material, which is available to authorized users.

## Background

### Background

Plants are central to almost all life on the earth, providing nourishment and protection for organisms ranging from bacteria to large animals [1–3]. Humans derive food, medicines and a number of ecosystem services such as air purification, origin and recharge of water bodies, nitrogen fixation, cycling of nutrients as well as many more other products from plant biodiversity [4–6]. The perception and relative importance of useful plants are related to cultural factors such as human behaviour, social and economic constraints, and several others [7, 8].

Nature and human culture converge on many levels that span values, beliefs and norms to practices, livelihoods, knowledge and languages [1, 9]. There exists a mutual feedback between cultural systems and the environment, with a shift in one often leading to a change in the other [10]. It has been suggested [11] that distinctions between social and natural systems are somewhat artificial and arbitrary. Traditional societies have interacted with biological diversity through adaptive and co-evolutionary processes for thousands of generations [12, 13]. This symbiotic relationship between biological and social systems helps in the hope of achieving a sustainable future [9, 14]. Maintenance of cultural diversity into the future, and the knowledge, innovations and outlooks it contains, increases the capacity of human systems to adapt and cope with change [7, 12].

Ethnobotanical studies are useful in documenting, analyzing and disseminating knowledge and interaction between biodiversity and human society, how diversity in nature is used and influenced by human activities [9]. Ethnoecological investigations document the knowledge on cultural interactions of people with plants and their environment. It also tries to find out how local people have traditionally used plants for various purposes and how they incorporated plants into their cultural traditions and religion [15–17].

Traditional management regimes and knowledge systems of forest resources have shaped forests throughout the world where materials from individual species are harvested in a sustainable manner [18]. The use of elaborate taboos, myths, folklore and culturally controlled systems, which bring coherence and shared community values to resource use and management are integral elements of traditional forest management systems [19]. The breakdown of many of these systems due to pressure of urbanization, cash economies and other socio-economic, political and cultural changes has resulted in the loss of forests and valuable species [17, 20, 21].

Ethiopia encompasses an amazing number of ecological Zones [8, 22] and plant species [23, 24]. Currently, however, the biodiversity of Ethiopia faces several threats. The main threats are government institutional capacity, population growth, land degradation, deforestation and weak management [22, 25]. As a result, habitats have been encroached or destroyed, diversity has been eroded, and livelihoods derived from biodiversity are threatened. Human activity has had disturbing impacts on forest resources and biodiversity [7, 26].

Historical sources in Ethiopia indicate that based on the potential climatic climax, some 40% of Ethiopia’s land area have originally been covered by closed forest [27, 28]. However, during the last century it has declined both in size and quality [29, 30]. By the early 1950s, high forests were reduced to 16% of the total land area, 8% in the 1960s, 4% in 1970s, by 1989 to about 2.7% and less than 2.3% in 1994 [28, 30, 31]. According to recent estimates by Reusing [32, 33], forest cover of Ethiopia was 1.41% in 1996 - 1997.

It has been estimated that 87% of the total land area above 1500 m a.s.l. was originally covered by dense forest [34], but now only 3% of the country is fully stocked with natural forest and that forest is disappearing at a rate of 7.5% per annum; the fastest rate of any country in the world [35]. A report indicates that the annual loss of the high forest area of Ethiopia is estimated between 150, 000 and 200, 000 ha [29], a rate at which in 15 years time the remnants of these high forests would be scattered patches in inaccessible areas [27, 36]. This enormous reduction in forest cover of the country has led to a marked increase in grass and degraded shrub vegetation and overall biodiversity erosion. The transformation is most advanced in the northern highlands of Ethiopia where the population has been concentrated and land has been cultivated for many centuries.

The high levels of dependency of the local community on agriculture (more than 90%) and high rate of population growth [37] have also accelerated the problems. Apparently, biodiversity resources along with their habitats are rapidly disappearing in many parts of the country [2, 38, 39].

To conserve the remaining natural forests of Ethiopia and the environment for the genetic resources and raw material for the industries, 58 National Forest Priority Areas (NFPA’s) covering an area of 3.6 million hectares have been selected [32, 40]. However, various studies indicate that protection of these NFPA has not been effective [25]. The NFPA failed to fully recognize the historical and customary rights and interests of local communities in forest products and forest lands. Local communities have frequently disregarded the boundaries established by the forestry sector on the notion that boundaries have violated their traditional access to and dependence on the forest resources. Management plans of the government are perceived by local communities as the state’s attempt to assert claims and rights, which do not acknowledge the interest and rights of the local people.

It has been suggested [41] that improving the management of the natural resources while providing ecological services and immediate economic needs are the major research and development challenges for the degraded areas of northern Ethiopia in particular and the drylands in east Africa in general. Thus, accommodating new conservation approaches such as participatory forest management can contribute significantly to mitigate the problem of forest destruction. The proper conservation of diverse habitats and genetic resources in countries like Ethiopia can only be achieved through a well-established system under which biological resources are sustainably exploited for immediate use and where species continue to evolve with the dynamic force of their habitats [26].

So far, studies on human forest interactions in dry Afromontane forests, including Hugumburda-Gratkhassu National Forest Priority Area, North-eastern Ethiopia, have been inadequate. Therefore, in view of the need to develop more effective approaches to conservation and sustainable utilization of forest resources in Hugumburda-Gratkhassu, an investigation of indigenous knowledge of the local community in conservation and utilization of forest resources was conducted. Certainly, the results of the study will give some information to plan appropriate intervention strategies for managing ecosystems in the area, and in regions in northern Ethiopia with similar problems.

In this study, we examined the following issues: (i) Which plant species are more important/exploited from the forest by the local inhabitants? What are the diverse uses obtained from each plant species? (ii) Why are there fewer trees now compared to the past? Who cut them or did they die, and why? (iii) What are the activities most destructive to the forest? (iv) Are there any traditional laws preventing people from harvesting forest products? (v) What appropriate measures should be taken to conserve traditional knowledge and plant diversity of the study area?

## Methods

### Description of the study area

**Fig. 1:**
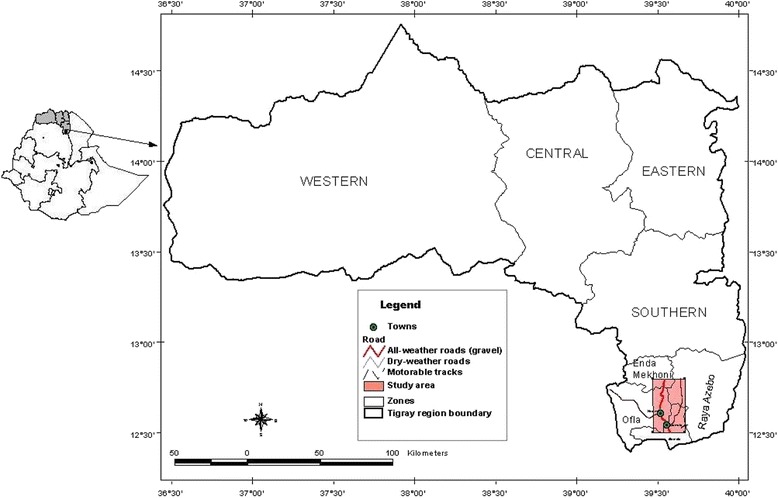
Map of the study area

Hugumburda-Gratkhassu National Forest Priority Area is located in North-eastern Ethiopia at about 600 km north of Addis Ababa and 160 km south of Mekelle, the capital of Tigray Regional State. It is located between 12^0^ 22′ and 12^0^ 42’N latitude, 39^0^ 28′ and 39^0^ 40′ E longitude (Fig. 1). Altitudes range from 1560 m to 2688 m above sea level.

**Fig. 2:**
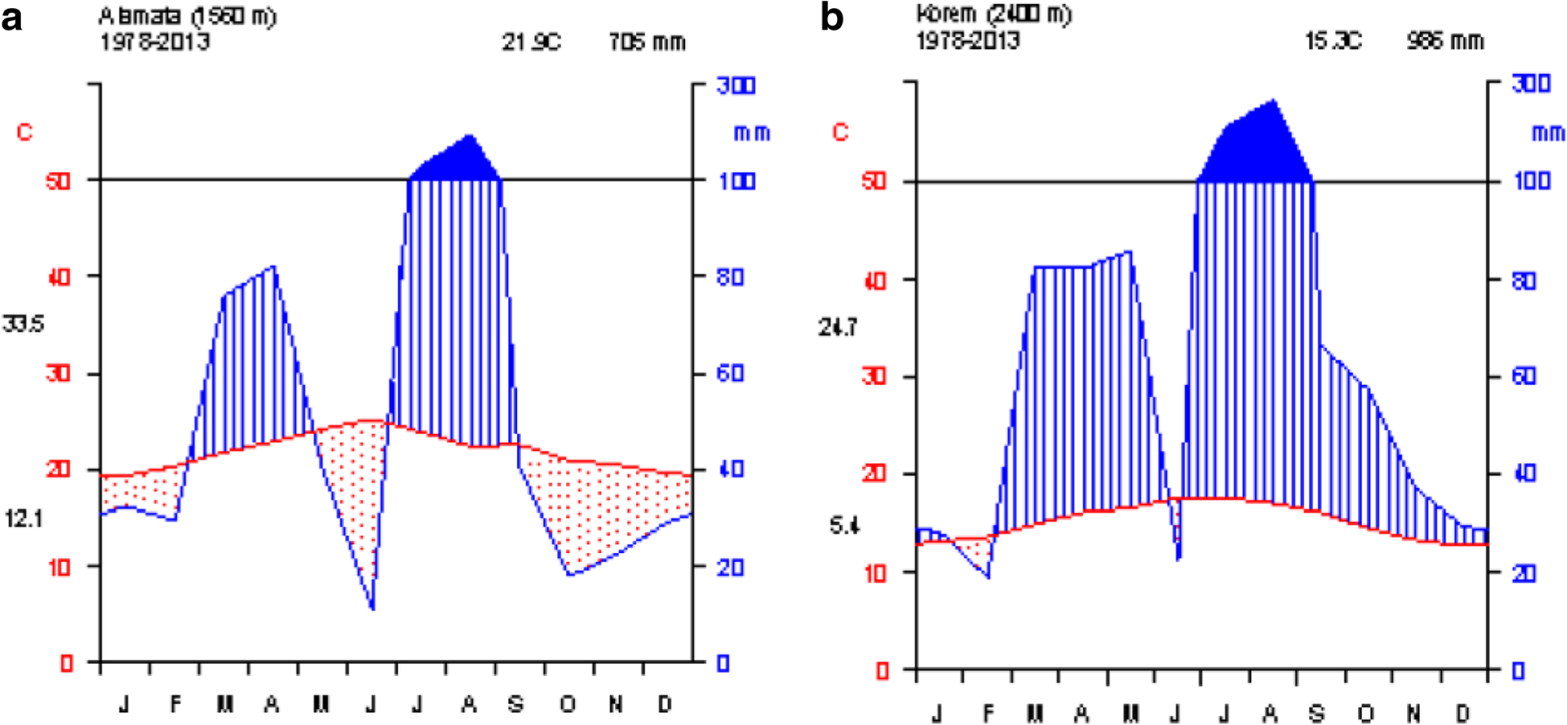
Climatic diagrams, (**a**) denotes Alamata and (**b**) denotes Korem. Data source: National Meteorological Service Agency, Ethiopia

There are two meteorological stations (Alamata and Korem) near the study area. Thirty five years (1978–2013) of meteorological data from these stations were acquired from NMSA, to describe the climate of the study area. Analysis of the meteorological data showed that the mean annual temperature for Alamata was 21.9 °C and the mean minimum and maximum were 12.1 and 33.5 °C, respectively (Fig. 2a). On the other hand, the mean annual temperature of Korem station was 15.3 °C with a mean minimum of 5.4 °C and a mean maximum of 24.7 °C (Fig. 2b). The hottest months are April and June, while coldness is from September to November. The mean annual rainfall for Alamata and Korem are 705 and 986 mm, respectively, but it varies greatly from year to year. Generally the study area has a unimodal rainfall pattern, with low rainfall from February to May and the main rainy season from June – September (Fig. 2).

Formerly, the area was covered with dense forest composed of different indigenous species. According to information obtained from local informants, the natural forest was exploited by an Italian concessionary named Montu Doro who installed sawmills at Hugumburda in 1950 with the permission of the then governor of Welo province. The forest was officially put under the auspices of the State Forestry Agency in 1965 [42]. Then in 1981 the area was identified as one of the National Forest Priority Areas (NFPAs). Boundary demarcation, which is the basis for the current management of the forest, was undertaken in 1993. Based on this demarcation, the project covers a total area of 21, 654.24 ha. Out of this 532.75 ha is plantation forest whereas the rest contains disturbed natural high forest, bushes, shrubs, agricultural plots and settlement areas.

There are 26,889 households within and around the forest boundary [43], out of which 5496 households are fully within the forest area and the rest (21,393) reside in the periphery of the forest [44].

### Methods for ethnobotanical data collection

#### Matrix ranking

A direct matrix ranking was conducted to discover local attitudes on species preference for multiple uses. During this work, the 46 most important tree and shrub species were selected among 102 tree/shrub species recorded from the study area [44], based on recommendations of local guides and key informants to determine the range of uses obtained from each tree and shrub species. In order to be consistent throughout the survey and for the purpose of comparison, the following use categories were adopted:

1. Farming tools
2. Construction material
3. Firewood
4. Medicine
5. Animal fodder
6. Tush (traditional incense)
7. Human food

**Fig. 3:**
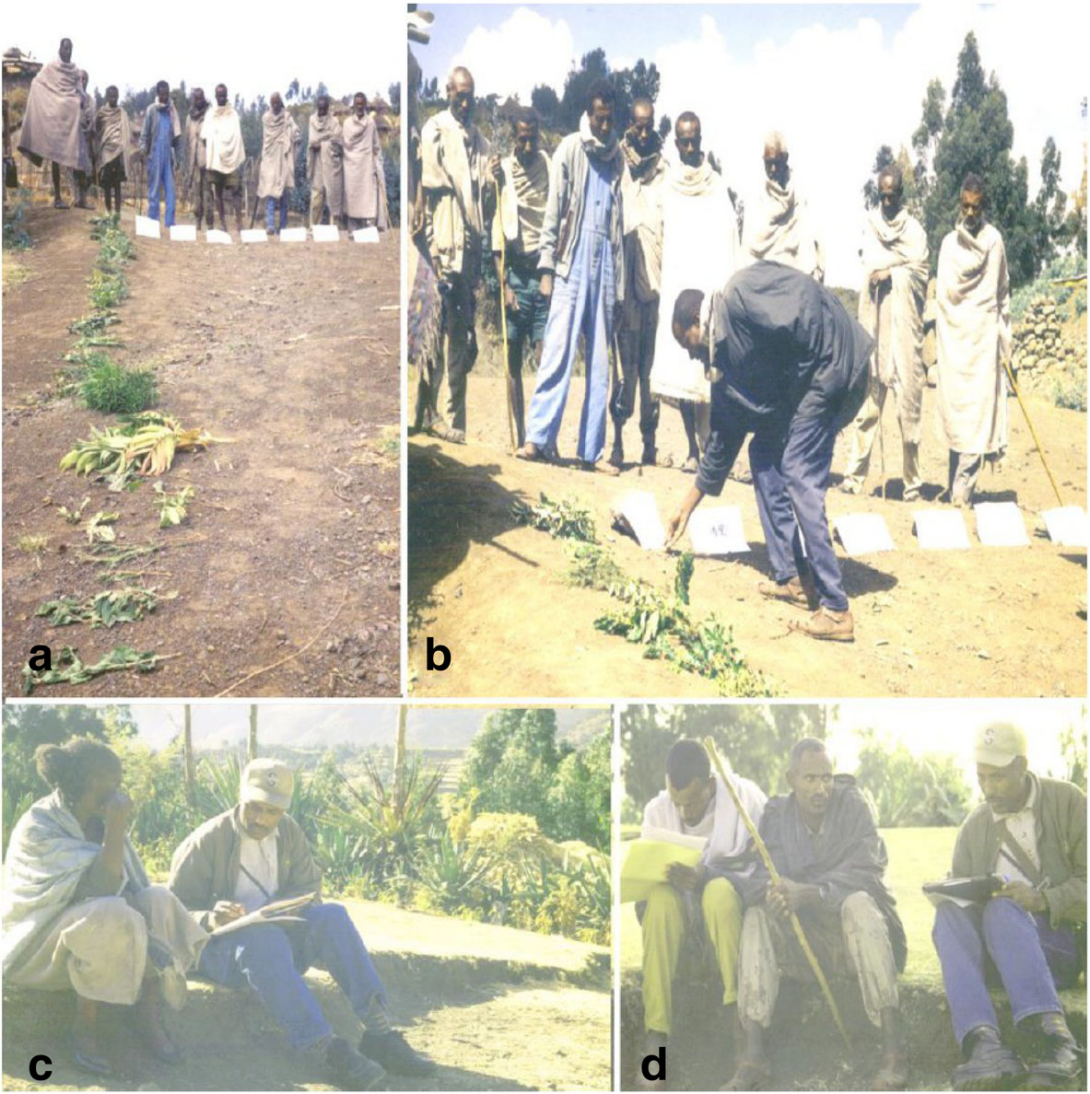
Local informants sharing their indigenous botanical knowledge. **a** Plant species displayed for direct preference ranking, (**b**) Informants giving value to each category of activity. Conducting an interview with local (**c**) female and (**d**) male informants concerning indigenous knowledge of conservation of forest resources

Samples of each plant collected before to the exercise were marked on the ground and placed along the rows and use categories along the columns as in Fig. 3. In the cells, the respondents placed a number of stones proportional to the importance of each species for each use (four for very good, three for good, two for fair and one for not good), moving across one entire horizontal row at a time to emphasize comparing the different uses of a single tree/shrub species. After the matrix was completed, the stones against each species were counted. A preference list of species was then made putting the species with the highest score first. This exercise was done in groups of 6 - 7 local key informants in each of the six groups formed during the exercise. Participants worked together and came to agreement on each score to be awarded a plant, since this approach can help to get effective results regardless of the constraint of time. Respondents for this exercise have been suggested and selected through local guides and leaders. This may facilitate greater openness during data collection, since informants feel that their participation has been locally endorsed.

#### Paired comparison

Activities supposed to be the major threat to degradation of the forest in the study area as perceived by local guides, literature and general observation of the researcher were adopted for this exercise. Accordingly, the following categories of activities were established:

1. Charcoal making
2. Construction material
3. Farming tools
4. Fuelwood collection
5. Hive making
6. Grazing
7. Forest fire

The numbers of pairs of activities were established as described by [9] from the relation n (n-1)/2 where n is the number of activities. In this case, following alphabetical arrangement, 21pairs were obtained serially, i.e., 1, 2; 1, 3; 1, 4; −-6, 7. The pairs were written on 21 pieces of paper, which were mixed together in a container. From the container, pieces of paper were picked one after another without replacement. The first pair to be picked was assigned serial number 1, the second 2, up to the last that was given serial number 21(see Additional file 1: Appendix 1).

The established randomized pairs were used to get the responses of individuals as described by [9, 45]. Each respondent was asked to select an activity that he considered being a major problem to management of the forest from each of the 21 established pairs. Scores for each respondent were recorded in a pairwise matrix (see Additional file 2: Appendix 2) and added together, and rank was assigned to each of the seven activities. A total of sixty individuals selected by purposive sampling based on recommendations of guides, local authorities, knowledgeable elders and development agents were involved in this exercise (see Additional file 3: Appendix 3).

#### Semi-structured interviews

Semi-structured interviews as described by [1, 9, 45] were used to obtain information from sixty local informants (Fig. 3). These were selected for interview after contacting the Woreda, Tabia and Kushet administrative units to address qualitative issues concerning community attitudes towards forest management and utilization, felt needs and to design an appropriate solution. During this exercise respondents were asked to respond to a checklist of open ended questions prepared beforehand (see Additional file 4: Appendix 4).

#### Voucher plant specimen collection and identification

During the data collection period, plant specimens were collected, pressed, dried properly and brought to the National Herbarium (ETH), Addis Ababa University for identification and authentication. The identification was done using the Flora of Ethiopia and Eritrea (FEE) [46–52] and by comparing with the authentic specimens in the ETH. Plant nomenclature followes FEE. The accuracy of identification was checked and confirmed.

### Data analysis

Responses recorded in a pairwise matrix during the paired comparison exercise were used to reveal the activities most threatening to the forest management. To be able to arrive at this, scores for similar cells for all respondents were added together and totals used to establish which activity was considered more threatening to the forest resources than others (see Additional file 2: Appendix 2). During matrix ranking stones placed in the cells were counted across the horizontal rows, which helped to place species in rank according to their scores. A descriptive statistical method such as percentage and frequency was employed to analyze and summarize the data. In addition, plant use values and the importance value index of the selected tree and shrub species were tabulated and analyzed statistically. Facilities in SPSS software (Version 20) and Excel spreadsheet were utilized to make simple calculations and determine proportions.

## Results

### Ethnobotanical investigation

The results obtained during the ethnobotanical investigation have been organized into three sections. The first section deals with the findings related to knowledge of local people on the value of plants. This is followed by a section, where threats to forest resources of the study area are presented. Finally, indigenous knowledge related to conservation of vegetation is presented.

#### Knowledge on the value of plants among local people

The relative uses of 46 tree and shrub species were assessed using direct matrix ranking and scoring. A preference list of species was then made putting the specimens with highest score first (Table 1).

**Table 1:** Total scores and ranks of trees and shrubs by direct matrix ranking exercise

Scientific name	Vernacular name (Tigregna)	Total score	Rank
*Olea europaea* L. ssp. *cuspidata* (Wall. ex G.Don.)	Awlie	969	1
*Balanites aegyptiaca* (L.) Del.	Bedano	826	2
*Dodonaea angustifolia* L.f.	Tahsos	790	3
*Ziziphus spina-christi* (L.) Desf.	Kunkura (Geba)	696	4
*Hagenia abyssinica* (Bruce) J.F.Gmel.	Habi	662	5
*Rhus glutinosa* A.Rich.	Tetaelo	661	6
*Myrica salicifolia* A.Rich.	Shihnet	660	7
*Acacia tortilis* (Forssk.) Hayne	Karora	653	8
*Acacia etbaica* Schweinf.	Seraw	602	9
*Allophylus abyssinicus* (Hochst.) Radlkofer	Meara	601	10
*Carissa spinarum* L.	Agam	597	11
*Psydrax schimperiana* (A.Rich.) Bridson	Tsehag	594	12
*Cassipourea malosana* (Baker) Alston	Keyh-om	593	13
*Rhus natalensis* Krauss	Atam	588	14
*Dovyalis abyssinica (*A.Rich.)	Mengolhats	586	15
*Erica arborea* L.	Hasti	581	16
*Podocarpus falcatus* (Thun) Mirb.	Zigba	574	17
*Juniperus procera* Hochst. ex Endl.	Tsihdi-adi	567	18
*Pavetta oliveriana* Hiern	Shumeja	565	19
*Acacia abyssinica* Hochst.ex Benth.	Chae	561	20
*Pittosporum viridiflorum* Sims	Mayliho	557	21
*Grewia mollis* A. Juss.	Reway	553	22
*Cupressus lusitanica* Mill.	Tsihdi-ferenji	533	23
*Nuxia congesta* R. Br. ex Fresen.	Tekarie	532	24
*Ficus sur* Forssk.	Shanfa	531	25
*Teclea simplicifolia* (Engl.) Verdoom	Salih	531	25
*Myrsine africana* L.	Kechemo	527	27
*Cadia purpurea* (Picc.) Ait.	Shilaen	526	28
*Olinia rochetiana* A.Juss.	Ale-ale	518	29
*Dovyalis verrucosa (*Hochst.) Warb.	Tuemtenay	518	29
*Calpurnia aurea* (Ait.) Benth	Hitsawits	517	31
*Rosa abyssinica* Lindley	Kaga	512	32
*Dombeya torrida* (J.F.Gmel.) P.Bamps	Buyak	485	33
*Ekebergia capensis* Sparm.	Kot	467	34
*Jasminum grandiflorum* L.	Tselim-habi	451	35
*Sageretia thea* (Osbeck) M. C. Johnston	Kenchelchele	444	36
*Euclea schimperi* (A.DC.) Dandley	Kuliow	430	37
*Maytenus undata* (Thunb.) Blakelock	At-at	429	38
*Ehretia cymosa* Thonn.	Tuwlaga	424	39
*Abutilon hirtum* (Lam.) Sweet	Necha	403	40
*Becium grandiflorum* (Lam.) Pic.serm.	Tebeb	395	41
*Meriandra bengalensis* (Konig ex Roxb.) Benth	Mesaguh	375	42
*Phytolacca dodecandra* L.Herit.	Shimti	369	43
*Otostegia integrifolia* Benth	Chi-endog	368	44
*Bersama abyssinica* Fresen.	Mirkuz-zibe	360	45
*Conyza hypoleuca* A.Rich.	Tsaeda-kotsilo	340	46

Based on the result, it was observed that almost all the species have at least one use, though most of the species are used for multiple purposes (Table 2). Traditionally, the local people have their own way of categorizing important plant species in the forest priority area according to the value they provided. As a result, from the total of 46 plant species, 13 (30.29%) plants (Table 2) were found to have three or more than three use values and 15 (34.95%) species had two use values while 18 (41.94%) species were selected to contribute for only one type of use. The species selected for different uses are given in the following way:

**Table 2:** List of plant species with three or more use values as identified by the local people

		Use categories							
	Species	FT	CM	M	T	AF	FW	HF	Total Uses
1	*Olea europaea ssp. cuspidata*	x	x	x	x	x	x	0	6
2	*Dodonaea angustifolia*	x	x	x	x	x	x	0	6
3	*Balanites aegyptiaca*	x	x	x	0	x	x	x	6
4	*Ziziphus spina-christi*	x	x	0	x	0	x	x	5
5	*Acacia tortilis*	x	x	0	0	x	x	0	4
6	*Hagenia abyssinica*	x	x	x	0	0	x	0	4
7	*Myrica salicifolia*	0	x	x	x	0	x	0	4
8	*Rhus glutinosa*	x	x	0	0	x	x	0	4
9	*Cassipourea malosana*	x	x	0	x	0	x	0	4
10	*Allophylus macrobotrys*	0	x	0	0	0	x	x	3
11	*Cadia purpurea*	0	x	0	x	0	x	0	3
12	*Dovyalis abyssinica*	0	0	x	0	x	0	x	3
13	*Podocarpus falcatus*	x	x	0	0	0	x	0	3

Note: *FT* Farming tools; *CM* Construction material; *M* Medicinal purposes; *T* Traditional incense (Tush); *AF* Animal feed; *FW* Firewood; *HF* Human food

X = has use value under the category; 0 = has no use value

##### Tree and shrub species preferred for preparation of farming tools

Nine species as indicated in Table 2 and also *Nuxia congesta, Pittosporum viridiflorum, Psydrax schimperiana, Teclea simplicifolia, Ekebergia capensis* and *Olinia rochetiana* were preferred for making farm tools. Farmer’s criteria for choosing the various species for different farming tools were: durability, high density, which does not cause irritation of the oxen necks, and lightness.

##### Tree/shrub species used as construction material

All species in Table 2 except *Dovyalis abyssinica*, as well as *Juniperus procera, Acacia etbaica, Acacia abyssinica, Ficus sur, Psydrax schimperiana, Rhus natalensis, Pavetta oliveriana, Cupressus lusitanica* and *Jasminum grandiflorium* were the most important tree and shrub species selected for construction purpose.

##### Species used for medicinal purposes

In addition to the six species indicated in Table 2 *Acacia etbaica, Myrsine africana, Calpurnia aurea, Phytolacca dodecandra, Otostegia integrifolia, Meriandra benegalenesis* and *Ehretia cymosa* were the most important species used as human medicine. The part used, the way it is used and the type of disease for which it is used vary from one to the other.

Local communities in the study area use different methods for preparation of harvested medicinal plants. They may be chewed, smeared, rubbed, infused in hot liquid, eaten raw, crushed and pounded, chopped and fumigated, as well as cut and smell. The plant parts used most frequently were leaves followed by roots and seeds. For instance leaves of *Phytolacca dodecandra* are used for abortion, to kill stomach parasites and to treat liver disease. Roots of *Acacia etbaica* are used for treating eye disease, joint pain and skin swelling, and the seeds of *Balanites aegyptiaca* are crushed, squeezed, mixed with water and drunk for treating diarrhea and other abdominal pain.

##### Tree and shrub species selected for traditional incense (“Tush”)

According to the group respondents, traditional incense (“Tush”) is important for women in order to keep a clean air with a good smell, and also in the houses as a repellant against insects. Accordingly, respondents identified eight species of plants for this purpose. These are the five species shown in Table 2 and *Euclea racemosa, Carissa spinarem* and *Rosa abyssinica.*

##### Species of plants preferred as animal feed

Within the group of interviewees 26 tree and shrub species were identified as being used as feed for animals. Besides the species in Table 2 *Maytenus undata, Nuxia congesta, Erica arborea, Pittosporum viridiflorum, Dombeya torrida, Pavetta oliveriana, Jasminum grandiflorum, Grewia mollis* and *Abutilon hirtum* were most preferred by local informants.

##### Species used as firewood

Firewood is in much demand by the communities in the study area. Even though collection of dry wood is prohibited, illegal collection is a common practice. Fabaceae with three species, *Acacia abyssinica, A. tortilis, A. etbaica* is the most widely used plant family for production of fuelwood in the study area.

##### Species used as human food

The forest includes a number of wild plants used as sources of food for local people. Four species shown in Table 2 as well as *Ficus sur, Rosa abyssinica, Carissa spinarum, Dovyalis verrucosa, Sageretia thea* and *Olinia rochetania* are among the wild plant species used as human food.

#### Paired comparison on the causes of vegetation destruction

A total of 60 key local informants have been involved in this exercise. It was found that 17 people (28%) consider removal of vegetation for construction material as the most destructive activity. The total score awarded to this activity was 268 (21.44%) of the total score (1260). Hive making was considered to be the least destructive activity with a total score of 91 (7.28%). Destruction for farming tools and related equipment, was ranked second while fuelwood collection and charcoal making were third and fourth, respectively (Table 3).

**Table 3:** Total score and ranking of the seven activities supposed to be the major threats to degradation of the forest

No	Activities	Total score	Percentage (%)	Rank
1	Charcoal making	203	16.2	4
2	Construction material	268	21.4	1
3	Farming tools	237	19	2
4	Forest fire	135	10.8	5
5	Fuelwood collection	225	18	3
6	Grazing	101	8.1	6
7	Hive making	91	7.3	7
	Total	1260	100	

#### Responses from semi-structured interviews

##### Perception of the local community on the trend and importance of vegetation cover

Of the 60 local informants interviewed during semi-structured interviews 15 (25.05%) had lived 51-70 years in the vicinity of the forest, 23 (38.4) 31 -50 years, 14 (23.38%) 16 - 30 years, and 8 (13.4) up to 15 years. In general, the response to the question as to whether or not there have been changes in the vegetation cover and composition in the last 20-50 years was straightforward. All of the respondents indicated that there have definitely been changes, and the density of the tree and shrubby species has decreased tremendously. The majority of those interviewed understood and appreciated the linkages between vegetation cover, soil fertility and rainfall, and the subsequent effect of deforestation. They have tried to explain the importance of vegetation cover in maintaining environmental stability. The main reason given by the local informants for the loss of the vegetation cover includes: agricultural expansion to marginal lands for cultivation, mainly due to population pressure, which has been on the increase in the last few decades, introduction of sawmills, civil war, production of charcoal for sale and cutting tree/shrubs for firewood both for the market and home consumption.

##### Traditional Forest management practices in the study area

According to the respondents there are two methods of traditional forest management practices in the study area:

1. **“**Hizaeti” is practiced inside and outside the forest area. This is a common pool natural resource management system or a common property regime with a well-established set of rules. It implies a protected or safeguarded area which is protected by the beneficiaries. The local people govern their grazing area (Hizaeti) through their local by-laws (srit).
2. “Mewaya” Whereas Hizaeti are exclusively available for grazing by plow -oxen and totally closed from grazing from June to the end of September, ‘Mewaya’ are areas delineated for the grazing of other cattle. Contrary to the Hizaeti the Mewayas are grazed all year round. An example of rich tradition of safeguarding the trees inside Mewaya was observed in Mistay Ha of Hayallo Tabia (Ofla woreda) irrespective of the cattle interference throughout the year. Even though cutting of trees and shrubs, beside the grazing of cattle, is not allowed, due to a loose application of rules and regulations selective cutting of trees is common in the forest area.

## Discussion

Traditional societies have interacted with biological diversity through adaptive and co-evolutionary processes for thousands of generations [12, 13, 16]. Documenting and maintenance of cultural diversity into the future, and the knowledge, innovations and outlooks it contains increase the capacity of human systems to adapt and cope with change [7, 12]. Findings of the study indicate that the traditional concepts about plants are tied up with use of plants. A community that is dependent on particular resources for its survival generates a very deep sympathy for the pattern of variation in these resources [53]. Indigenous people throughout the world have their own distinct linguistic, cultural values and beliefs [54]. Similarly, the people of Ethiopia are knowledgeable about the names and classification of their environment, plants in their surroundings, and their value for the local people, which they have gained orally from generation to generation [2, 8, 55]. Similarly, local people of the study area have a diverse knowledge on plant use and forest management practices.

The result obtained from the direct matrix ranking exercise (Table 1) showed that 20 out of the 46 species of trees and shrubs compared were found to have highest scores and rank, indicating that these species are the most important and exploited tree or shrub species used by the local communities for multiple purposes in the study area. Thus, any plantation and enrichment activities in the study area should take into consideration to prioritize these highly ranked species.

The paired comparison exercise revealed logging for construction materials to be the major threat to Hugumburda-Gratkhasu state forest due to cutting of large volume of wood for construction of churches, health centers, schools, and new houses. *Juniperus procera, Olea europaea ssp. cuspidata, Rhus glutinosa, Ficus sur, Hagenia abyssinica, Cassipourea malosana* and *Acacia etbaica* are the most selected and exploited plant species for construction purposes. Seventeen respondents, 28% of all respondents ranked it first among the seven activities considered threats to the forest. This result is similar to other studies in different parts of Ethiopia [56] in general and Tigray in particular where the style of house construction requires the use of a large amount of wood [57].

All houses in Ethiopia particularly in the rural areas accounting for 85% of the population, and in the majority of urban areas are still made of wood as the major construction material derived from forest [56]. Similarly, the Loita Maasai people of Kenya used forest plants to construct their houses [58].

Selective logging of plants for farming tools and related tools is also severely destructive due to presence of a high population around and within the forest area. It was also observed that fuelwood collection for household consumption and for selling is another destructive activity, and the general reasoning was that all households in the area use fuelwood. A similar study made in Kafta-Humera [59] indicated, that fuelwood collection in the area is becoming the main problem for the existence of the forest, which showed fuelwood to be the main forest conservation problem in the area.

Another activity with high score was charcoal making, and the reason given was, that the process involves clear cutting and consumes large volume of wood. Forest fire, grazing and hive making are those activities that were considered as least destructive to the natural forest. The reasons as to why fire was scored a less destructive activity among the seven factors was that, fire was not common in the study area. Only seven respondents (11.7%) considered it as the most destructive, if it occurs. Fire hazard was observed in other studies [60, 61] to be the major threat to forest resources. However, the results obtained during this study do not reflect this reality. Fire hazard is grouped among the three least dangerous activities to forest resources together with grazing and hive making. Grazing was considered as less destructive. But 10% of the total respondents consider it to be the most destructive due to the destruction of sapling or seedlings which in turn affects regeneration capacity of the forest. Another activity considered less destructive was beehive making, as it was not common practice by all local people, and being selective to some specific tree species. Respondents also pointed out that beekeepers were not necessarily constructing new hives every year. This result is in agreement with another study in Dess’a forest, which indicates that grazing and beehive making are the activities that harm the natural forest least [52, 61].

## Conclusions

The survey showed that the forest is dominated by small sized tree and shrub species in a secondary stage of development, indicating that the forest was heavily exploited and affected in the previous periods, but good regeneration is in process at the present time. Though strong rules governing utilization of forest resources are in place, due to lack of awareness in the surrounding communities, illegal collection of fuelwood for sale and household consumption, cutting of live trees for construction, farming tools and other uses are common in the study area. The surrounding communities strongly object to the prohibition of collecting dry timber for building purposes and live trees for farming tools. Therefore, to improve the natural diversity and structure of the forest, to minimize the influence of the surrounding communities and to utilize the forest resources sustainably for present and future generations the following recommendations are forwarded:

1. A policy of no use can bring greater risk to an ecosystem where communities depend on the resources. The hostility caused by cutting off these resources can be an extremely risky strategy. Thus, the old tradition of isolating forests from the community has to be avoided and attitudinal changes must be brought about so the community feels that the forest is theirs.
2. Survival of protected areas depends on the support of local communities, rather than on fences, fines, or even force. In order to develop a trust between the local people and the Forestry Administration, the basic needs and traditional rights of the communities over the use of forest resources should be recognized. The much-needed positive attitudes towards forest protection and development can only be obtained from the rural communities through the development of a genuine benefit sharing mechanism. Thus, community participation is quite important to maintain and sustainably manage the forest resources.
3. Planting of multipurpose trees as described in the direct matrix ranking result in the fields of farmers land, introduction of agroforestry, sowing of good quality grass species and fodder plants and delineation of buffer zones can minimize pressure on the forest area.
4. We hope that this work contributes to the understanding of the phytosociology, ecology and management of the vegetation of Hugumburda-Gratkhassu National Forest Priority Area and stimulates further research on this remaining forest of the region.

## Additional files

Additional File 1: **Appendix 1.** A Pair wise ranking matrix for seven activities supposed to be the major threats to degradation of the forest. (DOC 43 kb)

Additional File 2: **Appendix 2.** Paired comparison field data collection form. (DOC 75 kb)

Additional File 3: **Appendix 3.** List of local informants contacted in the study area. (DOC 69 kb)

Additional File 4: **Appendix 4.** Schedule for semi-structured interview. (DOC 31 kb)
