# Supplementary material for: Human-Forest interfaces in Hugumburda-Gratkhassu National Forest Priority Area, North-eastern Ethiopia
Source: J Ethnobiol Ethnomed. 2018 Feb 23;14:17. doi: 10.1186/s13002-018-0218-7 (PMC5824611; doi:10.1186/s13002-018-0218-7)
Supplement: Supplementary file 1 — Appendix 1. A Pair wise ranking matrix for seven activities supposed to be the major threats to degradation of the forest. (DOC 43 kb) [file 13002_2018_218_MOESM1_ESM.doc]

**Additional file 1**

**Appendix 1.** Paired comparison field data collection form

| No | Order | Activities | Respondent No  1 2 3 4 5-------- |
| --- | --- | --- | --- |
| 1 | **1,7** | Charcoal making, Hive making |  |
| 2 | **4,7** | Forest fire, Hive making |  |
| 3 | **3,7** | Farm implements, Hive making |  |
| 4 | **5,7** | Fuelwood collection, Hive making |  |
| 5 | **1,2** | Charcoal making, Construction materials |  |
| 6 | **1,5** | Charcoal making, Fuelwood collection |  |
| 7 | **2,4** | Construction materials, Forest fire |  |
| 8 | **3,6** | Farm implements, Grazing |  |
| 9 | **4,5** | Forest fire, Fuelwood collection |  |
| 10 | **1,3** | Charcoal making, Farm implements |  |
| 11 | **2,7** | Construction materials, Hive making |  |
| 12 | **6,7** | Grazing, Hive making |  |
| 13 | **1,4** | Charcoal making, Forest fire |  |
| 14 | **2,6** | Construction materials, Grazing |  |
| 15 | **3,5** | Farm implements, Fuelwood collection |  |
| 16 | **1,6** | Charcoal making, Grazing |  |
| 17 | **3,4** | Farm implements, Forest fire |  |
| 18 | **2,3** | Construction materials, Farm implements |  |
| 19 | **4,6** | Forest fire, Grazing |  |
| 20 | **5,6** | Fuelwood collection, Grazing |  |
| 21 | **2,5** | Construction materials, Fuelwood collection |  |
