# Supplementary material for: Human-Forest interfaces in Hugumburda-Gratkhassu National Forest Priority Area, North-eastern Ethiopia
Source: J Ethnobiol Ethnomed. 2018 Feb 23;14:17. doi: 10.1186/s13002-018-0218-7 (PMC5824611; doi:10.1186/s13002-018-0218-7)
Supplement: Supplementary file 2 — Appendix 2. Paired comparison field data collection form. (DOC 75 kb) [file 13002_2018_218_MOESM2_ESM.doc]

**Additional file 2**

**Appendix 2**. Basic data matrix for the paired comparison of management problems of the forest

|  | Activities | R1 | R2 | R3 | R4 | R5 | R6 | R7 | R8 | R9 | R10 | R11 | R12 | R13 | R14 | R15 | R16 | R17 | R18 | R19 | R20 |
| --- | --- | --- | --- | --- | --- | --- | --- | --- | --- | --- | --- | --- | --- | --- | --- | --- | --- | --- | --- | --- | --- |
| 1 | Charcoal making | 3 | 3 | 1 | 3 | 6 | 3 | **1** | **2** | 1 | 5 | 6 | 3 | 3 | 2 | 3 | 2 | 2 | 3 | 3 | 3 |
| 2 | Construction materials | 6 | 6 | 6 | 3 | 5 | 4 | 4 | 4 | 4 | 6 | 5 | 5 | 6 | 4 | 5 | 6 | 3 | 5 | 4 | 5 |
| 3 | Farm implements | 5 | 5 | 4 | 5 | 4 | 6 | 4 | 3 | 4 | 4 | 3 | 4 | 3 | 3 | 2 | 4 | 4 | 6 | 4 | 3 |
| 4 | Forest fire | 2 | 1 | 1 | 6 | 1 | 1 | 2 | 3 | 6 | 0 | 0 | 2 | 3 | 6 | 3 | 1 | 0 | 1 | 1 | 6 |
| 5 | Fuelwood collection | 4 | 4 | 5 | 3 | 3 | 4 | 3 | 6 | 3 | 3 | 2 | 3 | 5 | 4 | 4 | 5 | 3 | 3 | 1 | 2 |
| 6 | Grazing | 0 | 2 | 1 | 0 | 0 | 1 | 5 | 1 | 1 | 2 | 2 | 1 | 0 | 1 | 1 | 0 | 4 | 1 | 3 | 2 |
| 7 | Hive making | 1 | 0 | 3 | 1 | 2 | 2 | 2 | 2 | 2 | 1 | 3 | 3 | 1 | 1 | 3 | 3 | 5 | 2 | 5 | 0 |
|  |  |  |  |  |  |  |  |  |  |  |  |  |  |  |  |  |  |  |  |  |  |
|  |  |  |  |  |  |  |  |  |  |  |  |  |  |  |  |  |  |  |  |  |  |
|  | Activities | R21 | R22 | R23 | R24 | R25 | R26 | R27 | R28 | R29 | R30 | R31 | R32 | R33 | R34 | R35 | R36 | R37 | R38 | R39 | R40 |
| 1 | Charcoal making | 4 | 3 | 4 | 2 | 4 | 3 | 3 | 4 | 2 | 2 | 5 | 6 | 6 | 4 | 4 | 4 | 4 | 6 | 1 | 2 |
| 2 | Construction materials | 5 | 6 | 4 | 5 | 4 | 5 | 5 | 5 | 3 | 4 | 4 | 5 | 5 | 5 | 3 | 3 | 4 | 5 | 3 | 3 |
| 3 | Farm implements | 4 | 5 | 4 | 5 | 5 | 6 | 4 | 4 | 4 | 5 | 6 | 3 | 3 | 4 | 3 | 1 | 2 | 3 | 2 | 2 |
| 4 | Forest fire | 2 | 3 | 1 | 2 | 1 | 1 | 2 | 2 | 2 | 1 | 2 | 2 | 1 | 3 | 5 | 3 | 3 | 2 | 2 | 1 |
| 5 | Fuelwood collection | 4 | 3 | 6 | 6 | 4 | 3 | 4 | 3 | 3 | 3 | 3 | 4 | 4 | 3 | 4 | 6 | 6 | 2 | 3 | 4 |
| 6 | Grazing | 1 | 0 | 1 | 1 | 2 | 2 | 3 | 3 | 6 | 6 | 0 | 0 | 2 | 0 | 0 | 2 | 0 | 0 | 5 | 5 |
| 7 | Hive making | 1 | 1 | 1 | 0 | 1 | 1 | 0 | 0 | 1 | 0 | 1 | 1 | 0 | 2 | 1 | 2 | 2 | 3 | 6 | 4 |
|  |  |  |  |  |  |  |  |  |  |  |  |  |  |  |  |  |  |  |  |  |  |
|  |  |  |  |  |  |  |  |  |  |  |  |  |  |  |  |  |  |  |  |  |  |
|  | Activities | R41 | R42 | R43 | R44 | R45 | R46 | R47 | R48 | R49 | R50 | R51 | R52 | R53 | R54 | R55 | R56 | R57 | R58 | R59 | R60 |
| 1 | Charcoal making | 3 | 3 | 1 | 4 | 6 | 5 | 5 | 4 | 1 | 2 | 6 | 4 | 4 | 4 | 4 | 3 | 2 | 4 | 3 | 4 |
| 2 | Construction materials | 5 | 4 | 5 | 4 | 4 | 3 | 5 | 3 | 3 | 4 | 5 | 4 | 5 | 6 | 5 | 4 | 4 | 4 | 4 | 5 |
| 3 | Farm implements | 2 | 5 | 6 | 2 | 3 | 3 | 2 | 5 | 5 | 3 | 3 | 5 | 4 | 5 | 6 | 5 | 5 | 5 | 3 | 5 |
| 4 | Forest fire | 6 | 1 | 3 | 3 | 3 | 2 | 2 | 6 | 2 | 2 | 2 | 1 | 1 | 1 | 2 | 3 | 1 | 2 | 3 | 2 |
| 5 | Fuelwood collection | 4 | 6 | 4 | 2 | 4 | 2 | 5 | 1 | 3 | 4 | 4 | 5 | 4 | 3 | 3 | 4 | 6 | 4 | 5 | 4 |
| 6 | Grazing | 1 | 2 | 1 | 0 | 1 | 6 | 2 | 0 | 1 | 5 | 1 | 1 | 3 | 2 | 1 | 1 | 2 | 1 | 2 | 1 |
| 7 | Hive making | 0 | 0 | 1 | 6 | 0 | 0 | 0 | 2 | 6 | 1 | 0 | 1 | 0 | 0 | 0 | 1 | 1 | 1 | 1 | 0 |
