# Supplementary material for: Human-Forest interfaces in Hugumburda-Gratkhassu National Forest Priority Area, North-eastern Ethiopia
Source: J Ethnobiol Ethnomed. 2018 Feb 23;14:17. doi: 10.1186/s13002-018-0218-7 (PMC5824611; doi:10.1186/s13002-018-0218-7)
Supplement: Supplementary file 3 — Appendix 3. List of local informants contacted in the study area. (DOC 69 kb) [file 13002_2018_218_MOESM3_ESM.doc]

Additional file 3

Appendix 3. List of local informants contacted in the study area

| **No** | **Name** | **Sex** | **Age** | **No** | **Name** | **Sex** | **Age** |
| --- | --- | --- | --- | --- | --- | --- | --- |
| 1 | Kebede Demeke | M | 31 | 31 | Hiluf Berhe | M | 38 |
| 2 | Jemal Kahsay | M | 38 | 32 | Brhahe Aleme | M | 40 |
| 3 | Kalaeita Reda | F | 40 | 33 | Berihun Fantaye | M | 42 |
| 4 | Tsehaytu Wondemu | F | 38 | 34 | Kebede Aletay | M | 62 |
| 5 | Beri Yasin | F | 37 | 35 | Zewde Abera | F | 35 |
| 6 | Endeshaw Adhana | M | 60 | 36 | Hadis Hadhana | M | 49 |
| 7 | Ebrahim Senbetay | M | 39 | 37 | Hagos Bogale | M | 70 |
| 8 | Reda W/gebreal | M | 68 | 38 | Hadis Amare | M | 40 |
| 9 | Halefom Berhe | M | 32 | 39 | Belay Abebe | M | 56 |
| 10 | Nuru Adem | M | 30 | 40 | Giday Molla | M | 30 |
| 11 | Ali Hage | M | 43 | 41 | Abrha Habe | M | 61 |
| 12 | Bere Mesele | M | 47 | 42 | Zinabu Terefe | M | 38 |
| 13 | Baraki Tadeg | M | 30 | 43 | Chekol Kahsay | M | 53 |
| 14 | Reda Kebedew | M | 50 | 44 | Kahsay Alemu | M | 48 |
| 15 | Teib Zeinu | M | 28 | 45 | Hagazi Tuemay | M | 30 |
| 16 | Mohamed Nigusse | M | 55 | 46 | Ferden Bisetegn | F | 40 |
| 17 | Haile Mola | M | 39 | 47 | Taye Berhe | M | 52 |
| 18 | Hagos Yasin | M | 68 | 48 | Reda Chekol | M | 59 |
| 19 | G/yesus Kahsay | M | 45 | 49 | Menenu Reda | F | 23 |
| 20 | Mehari Hagos | M | 24 | 50 | Kasahun Wegahta | M | 58 |
| 21 | Adem Mohammed | M | 40 | 51 | Wegah Dagnew | F | 56 |
| 22 | Haile Abrha | M | 30 | 52 | Giday Tareke | M | 40 |
| 23 | Tadesse Girmay | M | 37 | 53 | Dereje Mehari | M | 46 |
| 24 | Hiluf Mekonnen | M | 49 | 54 | Berhe Zereu | M | 51 |
| 25 | Gebru Tegegne | M | 47 | 55 | Megos Chekole | M | 36 |
| 26 | Libase Abay | M | 53 | 56 | Nigusse Alemu | M | 48 |
| 27 | Mebrhatu Sheka | M | 88 | 57 | Berhe Derbew | M | 60 |
| 28 | Amare Kelelew | M | 46 | 58 | Negassi Misgun | M | 38 |
| 29 | Hiluf Berhe | M | 30 | 59 | Kassahun Desalegh | M | 26 |
| 30 | Adisu Berhe | M | 33 | 60 | Hiluf Gessesse | M | 42 |
