# Supplementary material for: Human-Forest interfaces in Hugumburda-Gratkhassu National Forest Priority Area, North-eastern Ethiopia
Source: J Ethnobiol Ethnomed. 2018 Feb 23;14:17. doi: 10.1186/s13002-018-0218-7 (PMC5824611; doi:10.1186/s13002-018-0218-7)
Supplement: Supplementary file 4 — Appendix 4. Schedule for semi-structured interview. (DOC 31 kb) [file 13002_2018_218_MOESM4_ESM.doc]

**Additional file 4**

Appendix 4. Schedule for semi-structured interview

**Objective**: - To collect data from farmers/community/about their knowledge/understanding/of natural resources management and their sustainable use.

1. Kushet________________ 2. Tabia______________ 3. Woreda___________

4. Name of respondent_________________________

5. Sex________________ 6. Age_________________

7 Have you and your family always lived in this area?

8. Was the tree cover different before, i.e. more cover or less?

9. If so, how long ago were this?

10. For what type of purpose you are exploiting the forest products? Are trees important to you and your community and in what way?

11.Which plant species are more important/exploited/from the forest?

12.Has any particular tree species completely disappeared?

13.Why are there fewer trees now? Who cut them or did they die, and why?

14. I) Do you plant any trees? ______________________________

2) Which species? _____________________________________

3) For what purpose(s)_____________________________________

15. Have you noticed any change in the weather, soil conditions, etc? Since tree cover decreased?

16. What do you think are the most destructive activities to the forest?

17. What measures do you take to control deforestation? Are there any traditional laws preventing

people from harvesting forest products?

18. What type of Government decision is in place to prevent deforestation and loss of biodiversity?

19. How do you compare traditional forest management practices with the current rules used in

managing the forest?

20 Are you satisfied with the current rules in managing the forest? Why?

21 Have you ever been involved in making suggestions or decisions towards forest management?

22 What do you suggest has to be done in the future to improve the vegetation in

this area?
